# Multidisciplinary team training in postpartum hemorrhage: impact on the use of blood products

**DOI:** 10.61622/rbgo/2024rbgo67

**Published:** 2024-09-18

**Authors:** Carina Bauer Luiz, Ana Lúcia Letti Müller, Cristiano Caetano Salazar, Teresinha Zanella, Gabriel Cardozo Müller, Amanda Vilaverde Perez, Mariana Sbaraini, Maria Lucia Oppermann, Janete Vettorazzi

**Affiliations:** 1 Hospital de Clínicas de Porto Alegre Porto Alegre RS Brazil Hospital de Clínicas de Porto Alegre, Porto Alegre, RS, Brazil.; 2 Faculdade de Medicina Universidade Federal do Rio Grande do Sul Porto Alegre RS Brazil Faculdade de Medicina, Universidade Federal do Rio Grande do Sul, Porto Alegre, RS, Brazil.

**Keywords:** Postpartum hemorrhage, Blood transfusion, Maternal death, Patient care team, Mentoring

## Abstract

**Objective:**

Compare the number of puerperal women submitted to blood transfusion before and after the implementation of a care protocol for postpartum hemorrhage (PPH) with multidisciplinary team training.

**Methods:**

Cross-sectional study in a university hospital, analyzing births from 2015 to 2019, compared the use of blood products before and after the adoption of a PPH protocol with multidisciplinary training.

**Results:**

Between 2015 and 2019, there were 17,731 births, with 299 (1.7%) postpartum women receiving blood products and 278 postpartum women were considered for this analysis, 128 (0.7%) at Time 1 and 150 (0.8%) at Time 2. After the multiprofessional team training (T2), there was a difference in the complete use of the PPH protocol (use of oxytocin, misoprostol and tranexamic acid) (T1 = 5.1% x T2 = 49.5%, p≤0.0001). An individual categorized analysis revealed that, in the T2 period, there was lower use of blood component units per patient compared to T1 (Mann-Whitney, p=0.006). It should be noted that at T1 and T2, 54% and 24% respectively received two units of blood products. It is important to highlight that after the multidisciplinary team training for the PPH protocol, the goal of zero maternal death due to hemorrhage was reached.

**Conclusion:**

The adoption of a specific protocol for PPH, combined with the training of a multidisciplinary team, had an impact on the ability to identify women at high risk of hemorrhage, resulting in a decrease in the use of blood components.

## Introduction

Postpartum hemorrhage (PPH) is an important cause of maternal morbidity and mortality. It is the leading cause of maternal death in the world and the second most frequent cause in Brazil.^(1)^ Maternal death is a worldwide concern and one of the main problems of public health in Brazil, where the Maternal Mortality Ratio (MMR) reached the rate of 107,53 maternal deaths per 100,000 live births (LB) in 2021.^(2,3)^ The World Health Organization (WHO) considers rates below 20 maternal deaths per 100,000 LB to be acceptable, correlating lower rates with better quality of health services.^(4)^

Blood transfusion therapy is an important ally in the management of PPH, especially in women without clinical response to initial volume replacement.^(5,6)^ Blood transfusion is done in extremely severe cases and aims to increase patient survival while seeking diagnosis and/or effective treatment.^(7)^ It is considered an important indicator of severe obstetric morbidity and with the emergence of the Joint Commission International (JCI) protocols, the indicators of quality and safety in perinatal care were introduced, with the objective of performing statistical analysis of adverse perinatal outcomes, including blood transfusion.^(5,8,9)^

Protocols in obstetric practice have improved qualification in care. They lead to early recognition and intervention of PPH, decreasing blood transfusion rates and, consequently, decreasing maternal morbidity and mortality, in addition to improving communication and decreasing the stress level of professionals.^(10,11)^ Professional societies and global health agencies, including the *American College of Obstetrics and Gynecology* (ACOG), WHO and the *Royal College of Obstetrics and Gynecology* (RCOG) are in complete agreement on the need for PPH protocols.^(12)^ These protocols must have clear criteria for activation and early recognition of hemorrhage in order to provide a timely intervention. They should also propose drug scheduling, radiological and surgical interventions, and transfusion schemes.^(10)^ The California Maternal Quality Care Collaborative (CMQCC) defined stages of blood loss during PPH, with recommendations for interventions based on changes in vital signs or cumulative blood loss.^(12)^ Although PPH protocols are proven to be effective, work by Baird^(10)^ suggested that less than 70% of the maternities in the United States are using them in practice.

As health care has become increasingly complex, multidisciplinary education initiatives have been identified as promising strategies for more qualified, comprehensive and effective health care.^(13)^ The interprofessional learning environment, in addition to reinforcing the role of each profession, prepares for teamwork and develops positive attitudes for the benefit of the patients.^(14)^

Considering the relevance of prevention and early management of puerperal hemorrhage, the Pan American Health Organization (PAHO)/WHO, through the Latin American Center for Perinatology/Women’s and Reproductive Health (CLAP/MR), proposed the “Zero Maternal Death from Hemorrhage” strategy, with the goal of reducing maternal mortality from PPH by training health teams to be able to act in a coordinated and rapid manner when faced with emergency situations, especially in the first hour after delivery.^(15)^ At the Hospital de Clínicas de Porto Alegre (HCPA), this management protocol was called RED ALERT.

The aim of this study was to compare maternal outcomes in relation to the use of blood component transfusions before and after the implementation of a care protocol for PPH with multidisciplinary team training.

## Methods

A cross-sectional study was conducted with puerperal women admitted to HCPA between 2015 and 2019, a large public and university institution located in southern Brazil which attends around 3,500 births per year. In August 2017, multidisciplinary (doctors, nurses and nursing technicians from the obstetric center and the obstetric hospitalization unit) face-to-face training was carried out on how to use the institutional PPH Protocol. In this study, a comparative analysis was made between two periods: T1, the period before implementation – January 2015 to June 2017; and T2, the period after implementation of the PPH protocol and training of the multidisciplinary team – July 2017 to December 2019. All cases of women who received blood products during delivery and in the postpartum period were reviewed.

The institutional protocol for PPH care, called RED ALERT, consisted of a set of team actions, aiming at forecasting, managing and controlling PPH, as described below:

1. Classification of the hemorrhagic risk of patients admitted to the hospital for delivery assistance (Figure 1) and collection of a blood sample for laboratory exams, transfusion compatibility testing, and blood bag reserve, according to the identified risk;^(16)^
  **Figure 1:**
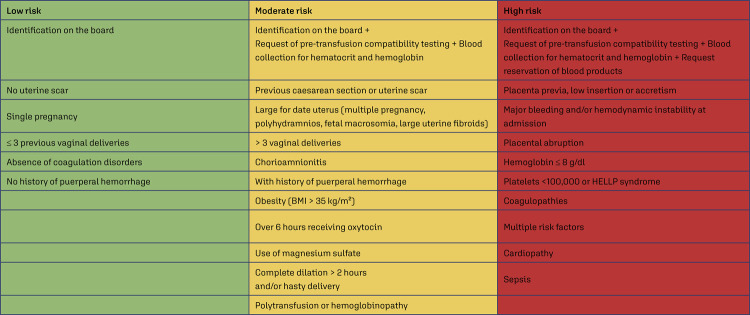
Obstetric hemorrhagic risk classification

BMI - body mass index; HELLP - hemolysis, elevated liver enzimes, low platelet count

Source: Adapted from Organização Pan-Americana da Saúde (OPAS). Recomendações assistenciais para prevenção, diagnóstico e tratamento da hemorragia obstétrica. Brasília (DF): OPAS; 2018 [citado 2022 Nov 7]. Disponível em: https://iris.paho.org/bitstream/handle/10665.2/34879/9788579671241-por.pdf. Quadro 3, Estratificação do risco para hpp; p. 11. ^(16)^
2. Early recognition of hemorrhagic emergency after prophylactic measures applicable to all patients (10 IU of oxytocin after umbilical cord clamping, placental delivery by controlled cord traction and uterine massage), and assessment of shock degree through comprehensive monitoring of vital signs (sensory, peripheral perfusion, blood pressure and heart rate). The criterion showing the most significant change was considered to estimate the volume of blood loss;^(17)^
3. Pharmacological management and volume replacement: additional oxytocin in solution, oral/rectal misoprostol, methylergometrine (if there are no contraindications), tranexamic acid and lactated Ringer’s solution. Medication administration was monitored using a checklist.;^(16)^
4. Collection of laboratory exams and triggering of coordinated between laboratory and blood bank (processing of test results and referral of blood products to the place where the patient is in the hospital - obstetric center, inpatient unit, operating room, intensive care unit). These actions were prioritized until the case was resolved;^(18)^
5. Continuation of the “4T” sequence of puerperal hemorrhage - Tone, Trauma, Tissue and Thrombin.^(16)^

In this analysis, women who received blood transfusions after vaginal or cesarean delivery with gestational age greater than or equal to 20 weeks of gestation were included. Women who underwent blood transfusion due to underlying hematological diseases (Sickle cell anemia or other previous severe anemia, Bernard Soulier syndrome, Marfan syndrome, thalassemia, stomach cancer, leishmaniasis, functional C4 deficiency, childhood leukemia, liver transplantation after childbirth, bone marrow aplasia, chronic thrombocytopenia, previous anticoagulation due to deep venous thrombosis and exchange transfusion) or who were transferred to HCPA from another institution after delivery were excluded.

The variables in this study were obtained from the participants’ electronic medical record using an instrument prepared by the researchers, which included age, ethnicity, education, body mass index, parity, characteristics and complications of current pregnancy, hospitalization and delivery, gestational age, induction and mode of delivery, history of postpartum hemorrhage, newborn data, hemorrhagic risk classification and complete use of clinical protocol for the management of PPH (drug and obstetric/surgical procedures), as well as which blood components were used and how many units, control exams performed and resulting complications, including mortality.

Data were processed using SPSS version 18.0 (Statistical Package for the Social Sciences; SPSS Inc., Chicago, IL, EUA®) after double entry and review of the database. Further analyses were made using the R software version 4.0.2 (R Core Team, 2021).^(19)^Quantitative variables are expressed as mean and standard deviation (± SD) or median (md) and interquartile range (25th to 75th percentiles), defined by the Shapiro-Wilk normality test. Comparisons between groups were conducted using the Student’s t test for independent samples or the Mann-Whitney test, when applicable. Qualitative variables are described by absolute (n) and relative (n%) frequencies, and possible associations between the categories analyzed were investigated using Chi-square test with adjusted residual analysis or Fisher’s exact test, with multiple comparisons corrected by the method of Bonferroni. The significance level adopted for all analyzes was set at 5%.

This study is part of the studies of the Quality Indicators and Assistance Safety in Obstetrics at HCPA, approved by the HCPA Research Ethics Committee under review number 3.038.148 and number of CAEE 80499417.8.0000.5327.

## Results

During the study period, a total of 17,731 births occurred at HCPA. Among these, 299 postpartum women (1.7%) received blood transfusions. Twenty-one of these cases (7.0%) were excluded due to pre-existing pathologies necessitating blood product use. The remaining 278 postpartum women, included in the study, were divided into two time series groups: T1, representing the pre-implementation period (January 2015 to June 2017, n=128); and T2, representing the period after the implementation of the PPH protocol and multidisciplinary team training (July 2017 to December 2019, n=150). The sociodemographic, obstetric, and perinatal characteristics of the participants included in the study are summarized in table 1. No statistically significant differences were found between the groups in terms of age (24 years in T1 vs. 26 years in T2, Mann-Whitney test, p=0.315) and BMI (29 kg/m^2^ in both groups, Mann-Whitney test, p=0.332). The majority of participants were of white ethnicity and had completed high school. There was a higher proportion of nulliparous women in T1 (12.5%) a higher proportion of multiparous women in T2 (50.7%). The absence of a history of PPH was slightly higher in T1 compared to T2 (67.5% vs. 56.2%, Fisher’s exact test, p=0.061). The rates of labor induction were similar in both groups (61.7% vs. 57.3%, χ2 with Yates continuity correction, p=0.463), as well as the rates of twin births, gestational age at delivery, mode of delivery, newborn weight, and APGAR scores (Table 2).

**Table 1:** Sociodemographic characteristics of postpartum women undergoing blood transfusion before and after multidisciplinary team training

Variables	T1 (n=128) n(%)	T2 (n=150) n(%)	*p-value
Maternal age (years)	24,00	26,00	0,315
md[P25–P75]	[20,00–33,00]	[21,00–32,00]	
(minimum- maximum)	(15,00–44,00)	(15,00–42,00)	
Ethnicity			
White	93(72,7)	112(74,7)	0,785
Non-white	35(27,3)	38(25,3)	
Education			
Illiterate/No Education	1(0,8)	1(0,7)	0,523
Incomplete elementary education	22(17,2)	26(17,3)	
Complete elementary school	19(14,8)	26(17,3)	
Incomplete high school	21(16,4)	17(11,3)	
Complete high school	49(38,3)	60(40,0)	
Incomplete higher education	11(8,6)	7(4,7)	
Complete higher education	5(3,9)	12(8,0)	
No information	0(0,0)	1(0,7)	
BMI (kg/m2)	29,02	29,39	0,332
md[P25–P75]	[24,54–32,81]	[25,58–33,90]	
(minimum- maximum)	(15,50–45,72)	(19,49–49,54)	
Parity			
Nulliparous	16(12,5)	3(2,0)	0,001
Primiparous	62(48,4)	71(47,3)	
Multiparous	50(39,1)	76(50,7)	

n - absolute frequency; n(%) - relative frequency; md - median; P25–P75 - interquartile range (25th and 75th percentiles); BMI - body mass index ( kg/m^2^); T1 - Time 1, January/2015 – June/2017; T2 - Time 2, July/2017 – December/2019; p: statistical significance index; *Chi-square test with adjusted residual analyzes or Mann-Whitney test

**Table 2:** Obstetric and perinatal data of postpartum women undergoing blood transfusion before and after training of the multidisciplinary team

Variables	T1 (n=128)	T2 (n=150)	*p-value
Gestational age (weeks)	39,36	39,07	0,344
md[P25-P75]	[37,14-40,14]	[35,86-40,14]	
(minimum- maximum)	(18,14 – 42,00)	(19,14 – 41,43)	
Type of pregnancy			
Single	125 (97,7)	145(96,7)	0,895
Multiple	3(2,3)	5(3,3)	
Labor induction	79(61,7)	86(57,3)	0,463
Delivery route			
Vaginal	82(64,1)	89(59,3)	0,459
Cesarean section	46(35,9)	61(40,7)	
History of postpartum hemorrhage			
None	83(67,5)	82(56,2)	0,061
Newborn Weight (g)	3230,00	3165,00	0,379
md[P25–P75]	[2710–3645]	[2682–3515]	
(minimum- maximum)	(375–4480)	(260–4535)	
APGAR Score 1° minute – md[P25–P75]	8,00[6,00–9,00]	8,00[7,00–9,00]	0,411
APGAR Score 5° minute – md[P25–P75]	9,00[9,00–9,00]	9,00[8,00–9,00]	0,354

n - absolute frequency; n% - relative frequency; md - median; P25–P75 - interquartile range (25th and 75th percentiles); g - grams; T1 - Time 1, January/2015 – June/2017; T2 - Time 2, July/2017 – December/2019; p – statistical significance index; *Chi-square test with adjusted residual analyses, Student’s t-test for independent samples or Mann-Whitney test

Prenatal complications (systemic arterial hypertension, pre-eclampsia/eclampsia, diabetes mellitus, premature labor, placenta previa, intrauterine growth restriction, anemia, premature rupture of membranes, urinary tract infection and polyhydramnios) were marginally higher in T1 (64.8% *vs* 54.7%, χ2 with adjusted residual analyses, p=0.061). Furthermore, T2 had a higher frequency of pre-eclampsia/gestational eclampsia (7.2% *vs* 20.2%, χ2 with Yates continuity correction, p=0.027).

The implementation of the new PPH care protocol for patient classification facilitated the identification of 34.7% of the cases of hemorrhagic risk in T2 (p≤0.0001) and influenced the clinical management of the patients. The use of uterotonics and complements for the management of hemorrhage (78.7%), especially oral misoprostol (39.3%) and tranexamic acid (56.0%), was more frequent after the implementation of the new protocol (χ2 with Yates continuity correction, p=0.025, p≤0.0001 and p≤0.0001, respectively). Additionally, hemostatic procedures were marginally more frequent in T2 (38.7%) justified by a higher number of hemostatic sutures (14.7%), while the performance of hematoma drainage was more frequent in T1 (7.0%) (χ2 with Yates continuity correction, p=0.056, p=0.003 and p=0.0028, respectively). The hemoglobin rate measured before/during bleeding events was significantly lower in T1 (Table 3). After application of the PPH care protocol, lower frequencies of post-hemorrhagic complications, especially acute renal failure (χ2 with adjusted residual analyses, p=0.010), were found. There was no significant difference in the number of women who required admission to the Intensive Care Unit (ICU) (10.9% *vs* 8.0%, p=0.137) and undergoing hysterectomy (9.4% *vs* 12.0%, p=0.611), as well as in relation to coagulopathies, sepsis, cardiorespiratory arrest and shock (Table 3).

**Table 3:** Application of the clinical protocol for PPH and post-hemorrhagic complications in postpartum women

Variables	T1 (n=128)	T2 (n=150)	*p-value
Hemorrhagic risk classification			
None	128(100,0)	98(65,3)	≤0,0001
Green	0(0,0)	19(12,7)	
Yellow	0(0,0)	22(14,7)	
Red	0(0,0)	11(7,3)	
Medication management	83(64,8)	118(78,7)	0,025
Use of uterotonics			
Oxytocin	68(53,1)	91(60,7)	0,516
Misoprostol orally	10(7,8)	59(39,3)	≤0,0001
Rectal misoprostol	55(43,0)	83(55,3)	0,542
Methylergometrine	37(28,9)	59(39,3)	0,476
Use of tranexamic acid	6(4,7)	84(56,0)	≤0,0001
Hemostatic procedures	35(27,3)	58 (38,7)	0,056
Suture of perineal tears	4(3,1)	9(6,0)	0,809
Curettage	17(13,3)	23(15,3)	0,517
Hematoma drainage	9(7,0)	2(1,3)	0,004
Hemostatic suture	5(3,9)	22(14,7)	0,028
Bakri Balloon Tamponade	4(3,1)	11(7,3)	0,505
Ligation of uterine arteries	2(1,6)	3(2,0)	1,000
Lower hemoglobin rate (g/dL) - (md-dp)	6,16(1,20)	6,66(1,24)	0,001
(minimum- maximum)	(1,1 – 9,5)	(3,7 -0 12)	
Post-hemorrhagic complications	14(10,9)	22(14,7)	0,457
Coagulopathies	6(4,7)	8(5,3)	0,603
Acute kidney failure	4(3,1)	0(0,0)	0,010
Cardiorespiratory arrest	2(1,6)	1(0,7)	0,388
Sepsis	2(1,6)	3(2,0)	0,743
Hemorrhagic shock	9(7,0)	14(9,3)	0,650
Non-hemorrhagic complications	22(17,2)	22(14,7)	0,682
Hysterectomy	12(9,4)	18(12,0)	0,611
ICU hospitalization	14(10,9)	27(18,0)	0,137
Maternal death	3(2,3)	1(0,7)	0,613

n - absolute frequency; n% - relative frequency; md: median; P25–P75 - interquartile range (25th and 75th percentiles); SD - standard deviation; g - grams; dL - deciliter; ICU - intensive care center; T1 - Time 1, January/2015 – June/2017; T2 - Time 2, July/2017 – December/2019; p - statistical significance index; *Chi-square test with adjusted residual analyzes or Mann-Whitney test

**Figure 2:**
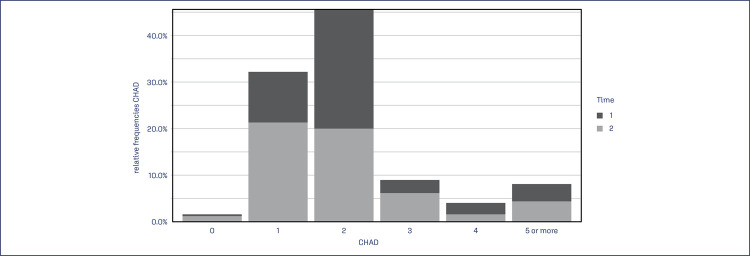
Use of CHAD units at Time 1 and Time 2

Regarding maternal deaths, there were 3 cases in T1, 2 of which had direct hemorrhagic causes (hypovolemic shock) and 1 indirect (septic shock + disseminated intravascular coagulation). After application of the protocol, there was no direct maternal death from hemorrhage, and the occurrence of 1 case in T2 was due to eclampsia with cardiorespiratory arrest and its consequences. The number of blood products used did not differ between the groups (Mann-Whitney, p=0.127) (Table 4), but individual categorized analyzes indicated that fewer units per patient were used in T2 compared to T1 (Mann-Whitney, p=0.006). Number of units used for plasma, platelets and cryoprecipitate did not differ between the temporal groups (Mann-Whitney, p=0.545, p=0.985 and p=0.241, respectively). Median and total packed red blood cells (PRBCs) units used differed between the two temporal groups (Mann-Whitney, p=0.046), and the categorized individual analysis indicated that T2 used a smaller number of PRBCs units per patient compared to T1 (Mann-Whitney, p=0.007) (Figure 2).

**Table 4:** Use of blood components

Variables	T1 (n=128)	T2 (n=150)	*p-value
Blood component units - md[IQR]			
Total units – n	620	538	0,127
Units per patient			
1	29(22,7)	58(38,7)	0,006
2	69(53,9)	51(34,0)	
3 or more	30(23,5)	41(27,4)	
CHAD units - md[IQR]			
Total units – n	351	322	0,046
Units per patient			
0	1(0,8)	3(2,0)	0,007
1	30(23,4)	59(39,3)	
2	72(56,2)	55(36,7)	
3	8(6,2)	17(11,3)	
4	7(5,5)	4(2,7)	
5 or more	10(7,8)	12(8,0)	
Plasma Units - md[IQR]			
Total units	83	74	0,545
Platelet Units - md[IQR]			
Total units	120	117	0,985
Cryoprecipitate units - md[IQR]			
Total units	66	25	0,241

n - absolute frequency; n% - relative frequency; md: median; IQR - interquartile range; SD - standard deviation; g - grams; T1 - Time 1, January/2015 – June/2017; T2 - Time 2, July/2017 – December/2019; CHAD - red blood cell concentrate; p - statistical significance index; *Chi-square test with adjusted residual analyzes or Mann-Whitney test

CHAD - red blood cell concentrate; Time 1 - January/2015 – June/2017; Time 2 - July/2017 – December/2019

## Discussion

The purpose of the study was to compare maternal outcomes in relation to the use of blood component transfusion before and after the implementation of a care protocol for PPH with multidisciplinary team training. The implementation was considered successful, achieving results in individual analyzes that indicated a lower use in the number of PRBCs units in T2 compared to T1. The consensus statement, Network for the Advancement of Patient Blood Management, Haemostasis and Thrombosis (NATA), recommends that hospitals that have obstetric units practice regular emergency exercises as a method of managing PPH, as, according to them, these are essential to maintain all professionals updated and alert for the early treatment of PPH.^(20)^

Preeclampsia has an estimated incidence of 3-5% of pregnancies in the world and 1.5% in Brazil. This condition is associated with a higher frequency of coagulopathies, which may require the use of platelets, even if there is no bleeding, or use of other blood products when there is hemorrhage. The increase in the transfusion rate at T2 can be explained by the concomitant significant increase in the incidence of preeclampsia at the same period.^(21)^

Our study found significance in the parity of women, both nulliparous and multiparous. As parity increases, muscle strength in the myometrium may decrease due to a reduction in existing collagen fibers; therefore, the higher the parity, the greater the probability of PPH.^(22)^ As for nulliparity at T1, we didn’t find in the literature a justification for its association as a risk factor for PPH, except a study in which 31% of women with PPH had no risk factors for this condition.^(23)^

Hamm et al.^(24)^ found non-significant trends toward increasing rates of PPH and blood transfusion after implementing a hemorrhage care package. This trend was observed for both subjective and objective definitions of PPH, which may be secondary to increased hemorrhage recognition, leading to increased estimation of blood loss and, therefore, of transfusion rates.^(24)^ In the present study, we identified an increase in the percentage of blood transfusions at T2 but a smaller number of PRBCs units used per patient, which may indicate a greater tendency in the identification of PPH and a more adequate management. With the implementation of the protocol, which established the inclusion of the classification of hemorrhagic risk, it was possible to identify 34.7% of the cases of hemorrhagic risk in T2, compared to none in T1 without this classification.

Drug treatments are essential in the management of PPH, especially when the reason is uterine atony. Therapeutic recommendations referring to uterotonics are diverse in the world literature; however, studies do not demonstrate superiority over one another, being important the simultaneous, and not staggered, use of medications. Furthermore, early use of tranexamic acid at the diagnosis of PPH is associated with lower maternal morbidity and mortality, with a reduction in the need for blood products and progression to more severe conditions.^(16-25)^ In the studied institution, the use of this medication was included as part of the protocol and was used in 56.0% of the women in T2 compared to 4.7% in T1, demonstrating the importance of training of the team. However, new actions are needed to increase adherence to tranexamic acid, as well as to the complete clinical protocol, since just over 50% and just under 50% of women, respectively, received the recommended drugs in T2 and T1. The most important action is the re-training of the team, which is always being renewed.

For Koch and Rattmann,^(26)^ if the hemorrhage persists after the administration of these uterotonics, other non-surgical strategies should be initiated (intrauterine tamponade balloon, for example) and as a last choice, surgical intervention.^(16-26)^ These literature data corroborate the findings of the present study, where the performance of hemostatic procedures was slightly more frequent in T2 due to higher number of hemostatic sutures.

Discussions about the best clinical practices among participants from the protocol training can remove intellectual and relational barriers between professionals, improve mutual understanding and promote learning.^(26)^ A study by Egenberg et al.^(27)^ in Tanzania showed a 47% reduction in blood transfusion rates after Kilimanjaro Christian Medical Center staff training. A similar result was seen in the Norwegian study by Egenberg et al.,^(27)^ where the prevalence of transfusions dropped from 3.4% to 2% after training. Future studies should be carried out to determine whether the effects of the educational initiative on the multidisciplinary team and increased maternal safety will be maintained in long term, as it can take several years to create and sustain changes in practices.^(11)^

Even with a reduction in the number of transfused PRBCs units, a significant reduction in ICU transfer rates and number of hysterectomies was not found. We consider this finding a consequence of the institution becoming a reference for the treatment of placental accretism in T2, which is a condition that increases the need for these procedures. With the institutionalization of the protocol, lower frequencies of post-hemorrhagic complications were observed, especially acute renal failure. Contrary to what is found in the literature, we didn’t find a reduction in the main morbidity rates.^(11)^ Baldvinsdóttir et al.^(28)^ found no improvement in PPH patient outcome regarding estimated blood loss, hemoglobin levels after transfusions, and PRBCs administered after the introduction of training for the obstetric team. However, they found a change in the clinical care of PPH which suggests that the team was better trained in its management and that they worked in a more standardized way when faced with it, ensuring safe venous access, fluid resuscitation, monitoring of vital signs, uterine massage and administration of uterotonics correctly, increasing maternal safety.^(28)^ As for the rate of maternal death related to hemorrhage, we found zero maternal deaths in T2 – in accordance with the institution’s PPH protocol aimed to have “zero maternal deaths” from hemorrhage, which is a very serious outcome that should be avoided at any time.

The implementation of the protocol for the management of PPH and the effect of the educational initiative on the number of transfused PRBCs units resulted in early recognition of hemorrhage, clarity of roles among the multidisciplinary team and also better understanding of the goals that must be achieved in the management of PPH.^(11)^ Finally, it should be noted that team training, simulation, and compliance auditing are essential resources to ensure that the protocols lead to a reduction in hemorrhage management, according to Federspiel et al.^(29)^

As a limitation of our study, we emphasize that it was conducted retrospectively and observationally, focusing on women experiencing Postpartum Hemorrhage (PPH) necessitating blood products. This methodology presents potential biases, including challenges in definitively establishing cause-and-effect relationships. The exclusion of women not requiring blood products introduces selection bias, potentially limiting the generalizability of our findings. It’s plausible that the institutional PPH management protocol may benefit other less severe groups, where blood products aren’t needed, but we didn’t explore this aspect in our study. We acknowledge that while our conclusions are significant for understanding the protocol’s effectiveness in a specific group, prospective studies are needed to further clarify the advantages of routine training in managing postpartum hemorrhage. Such studies could offer insights into how the protocol might enhance outcomes for a wider range of patients, including those with less severe PPH not requiring blood product intervention.

## Conclusion

In contemporary obstetric care, enhancing quality and mitigating maternal morbidity and mortality stand as top priorities, and implementing patient safety protocols is paramount. However, achieving full operational effectiveness in these protocols is often challenging. Such processes typically necessitate multidisciplinary teams, workflow adjustments, and shifts in institutional culture. Hence, our findings underscore the importance of specific management protocols and multidisciplinary team training in enhancing the identification of PPH risk in women. This approach optimizes blood product utilization and consequently reduces morbidity associated with PPH. Nonetheless, it’s crucial for these training initiatives to be ongoing, as a significant number of postpartum women with hemorrhage did not receive the complete protocol even after training.
